# Eligibility definitions and response rate accuracy in studies using routinely collected PROMs: a systematic review

**DOI:** 10.1007/s11136-026-04320-x

**Published:** 2026-06-25

**Authors:** Johanna Laine, Eila Kankaanpää

**Affiliations:** 1 Department of Health and Social Management, University of Eastern Finland, Kuopio, Finland; 2 Department of Strategy and Development, Wellbeing Services County of North Savo, Kuopio, Finland

**Keywords:** Patient reported outcome measure, PROM, Eligibility, Response rate, Routine collection, Health care

## Abstract

**Purpose:**

Patient reported outcome measures (PROMs) are increasingly used to assess treatment outcomes and quality of care. This systematic review examined how eligible populations and response rates (RRs) were reported in published studies using routinely collected PROMs.

**Methods:**

A systematic literature search in the PubMed, Scopus, Web of Science, Cinahl, PsycINFO and Cochrane library databases on Jan 15, 2024, and Feb 10, 2025, focused on studies employing routinely collected PROM data in research. Articles not related to a health care context were excluded. Definitions of eligibility were categorized as “all treated”, “all recruited” or “other”. RRs were obtained at baseline (BL) and the last follow-up (FU), and their accuracy was assessed according to the eligibility definitions.

**Results:**

From 3806 abstracts and 344 full-text articles, 92 articles (94 datasets) were included. Eligibility was defined as “all treated” in 26% of the studies and as “all recruited or “other” in 41% and 33%, respectively. Authors frequently fail to apply their own eligibility definitions in RR calculations, leading to incorrect RRs in 47% of datasets due to incomplete eligible populations or flawed denominator choices. Only 5% of studies clearly defined RRs. RR values varied widely. The systematic use of a defined eligible population was linked to a decline in average RRs over the FU.

**Conclusion:**

These findings suggest that inaccurate eligibility definitions and denominator use may bias RRs, limiting the reliability of PROM data. Although PROM datasets are increasingly used, data quality remains underdiscussed. This review highlights the need to improve the reporting of eligibility and RRs while using routinely collected PROMs. Protocol registration: PROSPERO CRD42023494821.

**Supplementary Information:**

The online version contains supplementary material available at https://doi.org/10.1007/s11136-026-04320-x.

## Abstract (plain language summary)

We studied surveys that ask patients how they feel about their health, symptoms, treatment and recovery. The findings are called patient-reported outcome measures (PROMs). Out of 92 studies, most surveys did not explain clearly who was included or provided responses. Only a quarter of surveys reported those included and only 5% clearly defined the proportion of people who returned responses. This could give a false picture of how patients feel about their health. To truly reflect patients’ health care views, surveys of PROMs need to address observed limitations.

**Supplementary Information:**

The online version contains supplementary material available at https://doi.org/10.1007/s11136-026-04320-x.

## Introduction

Greater demand for measurement of outcomes by regulators, accreditors, professional organizations, and payers has increased the need to implement patient-reported outcome measures (PROMs) in daily clinical practice [1] to assess treatment outcomes, the wellbeing of patients and the quality of care [2–4]. The rapid progression of technology and modern measurement methods has enabled the use of web-based electronic systems with real-time scoring and reporting of PROMs. These systems allow the widespread systematic implementation of electronic PROMs (ePROMs) under real-life clinical conditions in everyday practice for individual patients throughout a patient’s course of care [1, 5].

As the integration of PROMs into routine follow-up (FU) protocols has become increasingly common, the issue of low patient response has emerged as a more significant concern [6, 7]. PROM data have been used in various contexts, such as clinical trials and cohort studies, with differing data collection methods. Prospective cohort studies have reported significantly higher response rates (RRs) at baseline (BL), with 97% declining to 82% at 1 year, 74% at 1–2 years, and 61% at 2–5 years follow-up (FU), whereas registry-based studies report lower RRs (72%) at baseline and 64%, 57%, 47%, respectively [8]. Most registries exhibit considerable variability and a declining trend in RRs over time [9–11]. RR has been reported to decrease at follow-up (FU) as time passes and with repeated exposure to the same PROMs [8–10]. Loss to FU is common in cohort studies, resulting in potential bias and diminished statistical power [12]. Specific RR targets have been identified. In randomized clinical trials (RCTs) and cohort studies loss to FU of 5–20% have been shown to pose intermediate threats, and ≥ 20% serious problems with the validity of the results [13]. In the context of epidemiological studies, losses to FUs of 20–40% [12] or from 20 to 50% for FUs [13] have been suggested as acceptable. In most cases, these considerations have not been tested for validity [13]. While goals such as ≥ 90% reporting coverage and ≥ 80% RR are common, they are challenging to meet.

The number of eligible patients should encompass the entire target population and be determined based of the sampling frame, with RRs calculated for all eligible patients at baseline (BL) and during follow-up (FU) [14]. High coverage, defined as the proportion of eligible patients included, and consistently high RRs, the proportion of patients completing PROMs, are critical for data accuracy and reliability [8].

### Specific aims and main outcome(s)

We focused on research using PROM data collected in routine health care. The aim was to explore the reporting of eligible populations and RRs in peer reviewed articles. The primary research question was as follows:

How have authors defined eligible populations and calculated RRs of the routinely collected PROMs? We also studied the reported RRs, ranges and means and assessed the accuracy of the RR calculations.

## Methods

### Data sources

This systematic review followed a registered protocol (PROSPERO: CRD42023494821) [15]. This systematic review was conducted in accordance with the PRISMA 2020 guidelines [16], with the checklists provided in Online Resource_1. A comprehensive search strategy was developed in collaboration with an information specialist.

### Inclusion and exclusion criteria

Studies that used PROMs collected routinely in health care and reported RRs for PROM surveys were included. We included peer-reviewed published studies with no limitations on publication date or language that were conducted in all health care settings. Studies on adult patients 18 years and older of any ethnicity, sex, diagnosis, and treatment in health care context were included.

Observational studies, including prospective and retrospective longitudinal follow-up and cross-sectional studies, were considered for inclusion.

The reported PROMs had to provide at least one domain of a patient’s health. Patient-reported outcome (PRO) is defined as “any report of the status of a patient’s health condition that comes directly from the patient, without interpretation of the patient’s response by a clinician or anyone else. The outcome can be measured in absolute terms (e.g., severity of a symptom, sign, or state of a disease) or as a change from a previous measure” [17]. PROMs reflect the evaluation of a patient’s health status (physical, mental, and social functioning) from a patient’s own perspective, self-reported. We also included studies that used assisted PROMs or PROMs administered through interviews. PROMs reported by health care professionals (HCP) or proxies with no input from the patient were excluded.

Both in-clinic and remotely collected PROMs and all delivery formats for patient- or disease specific and/or generic PROMs were included regardless of the number of recalls and timing or duration of the data collection. Studies that focused solely on patient-reported experience measures (PREMs) and satisfaction with care received were excluded. Additionally, studies that passively collected health data by using health apps and wearable or medical devices were excluded.

Studies in which less than 10% of the study population was 17 years or younger were included if the other inclusion criteria were met.

The modified criteria for the inclusion or exclusion of articles are provided in Table 1 and in more detail in Online Resource_1, Supplement 3.

**Table 1:** The modified inclusion/exclusion criteria of the articles that used routinely collected PROM data

	Inclusion criteria	Exclusion criteria
Context	1. Patient- or disease specific and/or generic PROMs in health care context 2. PROMs providing at least one domain of patient’s overall-, physical- and/or mental health status, including symptoms, functional level and HRQoL 3. Routine collection of PROMs in routine clinical practice during the normal care processes and follow up	1. Unrelated non-health care context, e.g. population surveys 2. PROMs with no health status domains provided 3. Studies only on PREMs and satisfaction of care received, studies not related to the collection of PROMs in routine clinical practice and regular care processes and follow-up, PROMs collected in trials
Phenomena of interest	4. How is response rate (RR) calculated?	4. Response rate is not reported
Consept/administration	5. PROMs which can be self-administered and are self-reported 6. PROMs assisted by proxy (HCP/relative/ other person acting on behalf of the patient) and interview-administered or combination of these	5. Passively collected health data by using health apps and wearable or medical devices 6. PROMs reported by clinicians or other HCPs, proxy-reported with no input from the patient
Population	7. Adult patients 18 years and older (or less than 10% of the patients 17 years and younger) of any ethnicity, sex, diagnosis, or treatment	7. Patients under 18 years (more than 10% of the patients 17 years and younger)

*PROM* Patient Reported Outcome Measure, *PREM* Patient reported Experience Measure, *HRQoL* Health related Quality of Life, *HCP* Health care professional

### Search strategy and screening

The full search strategy is described in detail in Online Resource_1, Supplement 4. A systematic search was performed on January 15th, 2024, in the PubMed, Scopus, Web of Science, Cinahl, PsycINFO and Cochrane library databases. The search strategy was adapted for each included database. On February 10th, 2025, the searches were re-run for more recent publications. In addition, the reference lists of retrieved reviews were manually searched.

The identified publications were exported to Covidence (https://www.covidence.org/) for management and deduplication. In a pilot test, all authors individually assessed the inclusion/exclusion of 100 randomly chosen publications against the inclusion criteria (see Table 1). The process was standardized, with decisions discussed to ensure uniform selection process. Following a pilot test, two reviewers independently screened available titles and abstracts against the inclusion and exclusion criteria to ensure the inclusion of all relevant articles. The first author screened all the titles and abstracts and EK 46% of the potential articles. We had an additional reviewer for 54% of the titles and abstracts. Potentially relevant studies were then retrieved in full and judged for eligibility.

**Fig. 1:**
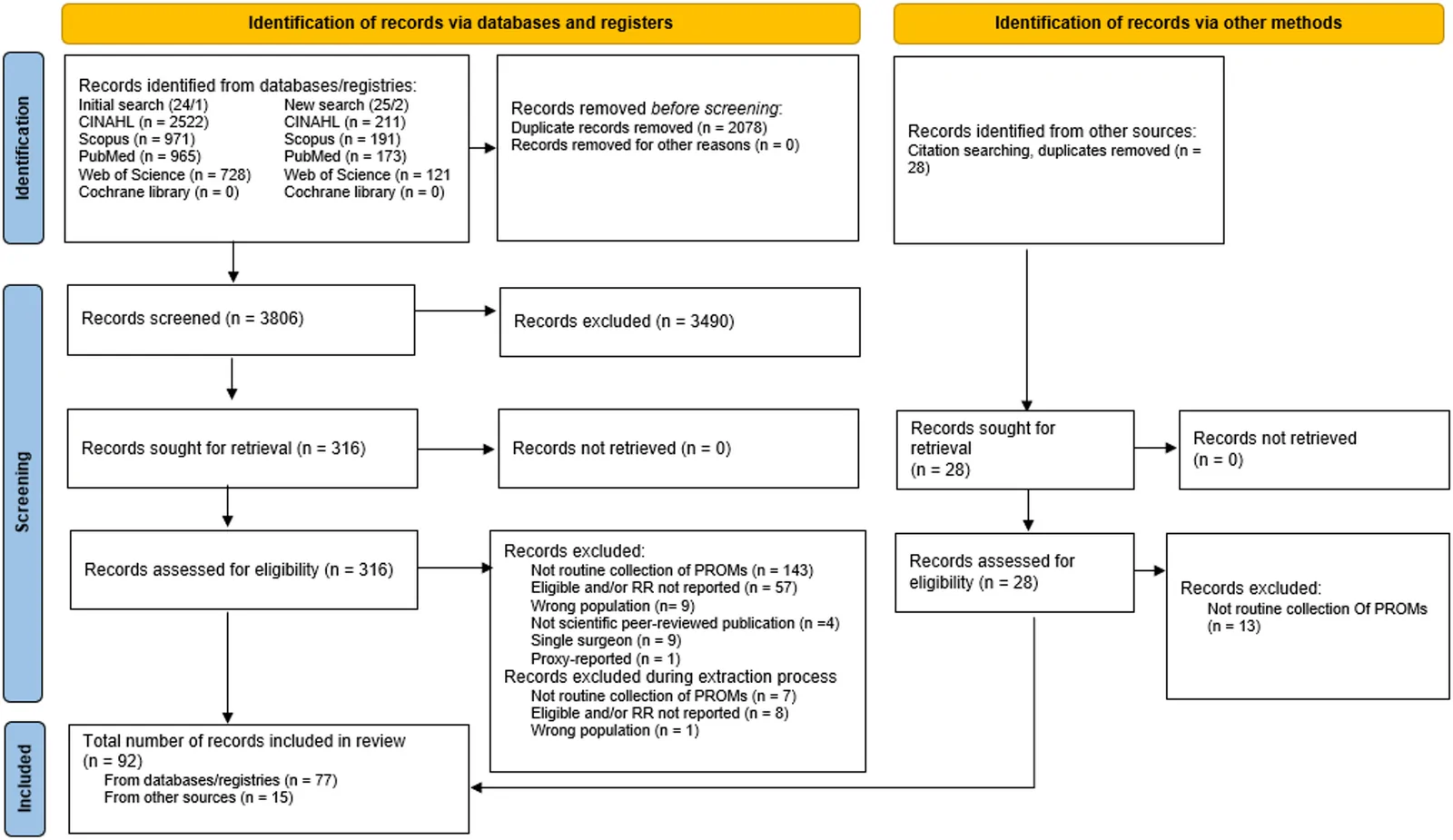
Search results and study selection and inclusion process [16]

Both authors individually screened all the full texts. Any disagreements that occurred between the reviewers were resolved through discussion. The corresponding authors of two original articles were contacted to obtain missing information relevant to the selection process. The reasons for exclusion were recorded and are presented in the Preferred Reporting Items for Systematic Reviews and Meta-analyses (PRISMA-2020_new_SRs) flow diagram [16] (Fig. 1).

### Data extraction

The data extraction tool was developed by the authors of this review. A pilot test with both authors individually extracting data from four randomly chosen publications was performed to ensure uniform extraction process. No disparities were found between authors. During the data extraction process, the data extraction tool was modified. The final version of the extraction tool with modifications is provided in Online Resource_1, Supplement 5.

The first author extracted data from all included articles, while the second author independently extracted data from a randomly selected 20% sample for cross-checking. Any discrepancies were discussed and resolved by consensus, and the process was fully documented, including key steps and both authors’ input.

### Data synthesis

A descriptive synthesis examines variations in year of publication, country of origin, study design, population characteristics, health care context (including clinical setting, type of care, organizational level) and the source of PROM data. We also summarized the details of the PROM data collection, including the number of surveys distributed over time and the number of questionnaires included per survey. The first author was responsible for data extraction and data synthesis.

First, we classified authors’ eligibility descriptions. The number of eligible patients should encompass the entire target population receiving the studied health care intervention (service, care). The RR should be calculated for all treated patients at BL and during FU and should be consistently used as a denominator at all time points [14]. Given that the definition of eligibility affects the calculation of RRs, we analyzed the calculation of RRs against the eligibility classification.

The first group consisted of studies correctly reporting eligibility as “all treated”. It was considered appropriate to exclude patients who had died, emigrated or revised during follow-up. Additionally, the exclusion of duplicates or other errors in reporting were accepted. The exclusion of participants for any other reason was deemed inappropriate. One study [18] with exceptionally long FU (15 years) and minimal loss (less than 0.5% annually) was classified as “all treated”. The second group, “all recruited”, contained studies that used eligible population patients recruited to a Clinical Quality Registry (CQR) or another specific database, such as a PROM platform, potentially representing a smaller subgroup. If coverage was 100% of the target population, it was classified as “all treated”. The third group, “other”, contained studies which incorrectly regarded baseline, follow-up, or baseline and follow-up respondents as the target population or used other inclusion criteria.

For the three eligibility categories, we analyzed how the RR was calculated. Only in the “all treated” group, if the authors followed standard methods for calculating RR, the RR was categorized as correctly calculated. In the other groups, “all recruited” or “other”, if the authors had used their definition of the eligible population as the denominator, we labelled RR denominator-consistently calculated. For example, if the number of patients in the data set was consistently used in RR calculations across all time points and RRs were reported separately for both BL and the last FU, the RR was considered denominator-consistently calculated irrespective the eligibility classification. If another figure was used as the denominator, we classified RR as incorrectly calculated. Additionally, if PROM surveys during follow-up were distributed exclusively to individuals who had responded at BL or at previous data collection time points, the RR was considered incorrect. In the “other” group, many studies did not present the number of respondents; thus, the accuracy of calculations could not be assessed. In all groups, inaccuracies related to incorrect denominators included missing reporting of FU time points, RRs reported only for individuals who completed both BL- and FU PROMs and the exclusion of patients without acceptable or clearly documented reasons. In some studies, the RR was not calculated.

We extracted the range and mean of the reported average RRs (percentages, %) of PROMs at baseline (BL) and at the last reported follow-up (FU) time point. When RRs were reported separately for several PROM questionnaires at the same time point, the highest available RR was selected. When RR percentages were not calculated, they were calculated by the review authors if data were available. We present the range and mean of reported average RRs by eligibility definition and RR calculation category. Differences in group means and RR attrition rates from BL to the last FU time of PROMs data collection were calculated and compared across groups. Given the substantial heterogeneity in FU durations (0 months–15 years), an analysis of average RRs across three FU time groups was performed to enhance data comparability (Online Resource_1). Throughout this study, the term dataset is used to refer to the populations reported in the articles. All the tabulated results are accompanied by a descriptive summary, and the detailed data extraction of all articles is provided in Online Resource_2.

## Results

### Included articles

In total, 5 884 articles were identified (Fig. 1). Duplicates were removed automatically (1 938) and manually (140). After screening 3 806 potential abstracts, 316 studies were identified for full-text retrieval. The selection process yielded 92 original publications in the analysis.

All the articles were published between 2011 and 2025, with 74% (68) from January 2020 onward. Most originated from the United States (33%) or Sweden (24%), followed by UK (10%), Denmark (8%), Norway (8%), other European (12%) or other (4%) countries. Two publications were multinational [19, 20]. Two studies [19, 21] included data from separate datasets, which were reported individually. The articles included are described in Table 2 and Online Resource_2.

**Table 2:** Characteristics of the included studies (*N* = 92, with 94 datasets) using routinely collected PROM data in research

	Studies/datasets (N)	Portion of total (%)
Year of publication	92	
2011–2019	24	26
2020	14	15
2021	10	11
2022	17	18
2023	14	15
2024	10	11
2025^a^	3	3
Country	92	
United States	30	33
Sweden	22	24
United Kingdom	9	10
Denmark	7	8
Norway	7	8
Other European country^b^	11	12
Other^c^	4	4
Multinational^d^	2	2
Study design	94	
Cross-sectional	20	21
Longitudinal	74	79
Setting	94	
Hospital or medical center	86	91
Ambulatory surgery center	1	1
Hospice	1	1
Other	6	7
Type of care	94	
Surgical or operative care	73	78
Acute, urgent or emergency care	8	9
Cancer or palliative care	7	7
Care of chronic illnesses	3	3
Preventive care or early intervention	1	1
Rehabilitation	2	2
Organizational level	94	
Specific to the disease or treatment	76	81
Specialty or clinic spesific	14	15
Organization wide	3	3
Other	1	1
Data source	94	
CQR	63	67
Local	16	25
National	42	67
National and local	2	3
International	3	5
Other	31	33
Another specific database (e.g., PROM platform)	5	5
EMR and/or another institutional or specific database	24	26
Routinely collected for research purposes	2	2

Percentages are rounded to the nearest integer

*CQR* Clinical Quality Registry, *EMR* Electronic medical record

^a^Studies published before February 10, 2025

^b^Other European country: The Netherlands, Switzerland, Finland, Germany

^c^Other: Australia, Japan

^d^Multinational: (1) Australia and Sweden, (2) Norway, Sweden and Denmark

The included datasets were either cross-sectional (20) or longitudinal (74) (Table 2). Most articles (78%) focused on surgical care, with data typically collected in hospitals or medical centers (91%) and often disease- or treatment-specific units (81%). Study populations were mainly orthopedic (65%), and PROMs were usually extracted from CQRs/databases (72%), mostly from national CQRs (67%).

Overall, 76 different PROMs (Online Resource_1, Supplement 6) were identified. The three most used PROMs are listed in Table 3. In majority of the studies (54%), both generic and specific PROMs were used.

**Table 3:** The three most used generic and specific PROMs

The type and name of the PROM^a^	Used in articles, *N* = 92	Percentage of total (%)
Generic		
EQ-5D/EQ-VAS	32	35
VAS/NRS	20	22
SF-12/VR-12	13	14
Specific		
HOOS/KOOS	21	23
OHS/OKS	12	13
PROMIS	8	9

*PROM* Patient Reported Outcome Measure

^a^Corresponding full terms of all PROMs available in Online Resource_1, Supplement 6

Table 4 provides an overview of routine PROM data collection (detailed in Online Resource_2). Most patients received the BL PROM survey before treatment or surgery (64, 68%). The cross-sectional data were mainly FU data (18 datasets, 90%), whereas the longitudinal datasets included both BL and FU (66, 89%) or FU (8, 11%) data.

**Table 4:** Details of the routine PROM data collection

	Cross-sectional (*N* = 20)		Longitudinal (*N* = 72)		All studies (*N* = 92)	
	Datasets (*N* = 20)	Percentage (%)	Datasets (*N* = 74)	Percentage (%)	Datasets (*N* = 94)	Percentage (%)
Timing of baseline (BL) survey						
Before treatment/operation	1	5	63	85	64	68
After diagnosis/first treatment/operation	1	5	1	1	2	2
After acute trauma/incident			2	3	2	2
No BL	18	90	8	11	26	28
Timepoints of PROM collection						
Baseline (BL) survey only	2	10			2	2
Follow-up (FU) survey only	18	90	8	11	26	28
Baseline and follow-up survey			66	89	66	70
The number of PROM questionnaires per survey (per time point)						
1	14	70	19	26	33	35
2		0	25	34	25	27
3	1	5	17	23	18	19
4	1	5	5	7	6	6
5	2	10	6	8	8	9
6 or more	2	10	2	3	4	4
Method(s) of administration						
Paper	7	35	13	18	20	22
Electronic	3	15	12	16	15	16
Paper and electonic	3	15	12	16	15	16
Paper and telephone			4	5	4	4
Electronic and telephone	1	5	2	3	3	3
Electronic and else	1	5			1	1
Paper, electronic and telephone			2	3	2	2
Paper and else			1	1	1	1
Paper, telephone and else			1	1	1	1
Paper, electronic, telephone and else			1	1	1	1
NR	5	25	26	35	31	33
The total number of surveys (all time points)						
1	20	100			20	21
2			34	46	34	36
3			20	27	20	21
4			6	8	6	6
5			4	5	4	4
6 or more			3	4	3	3
NR			7	9	7	7
The last follow-up (FU) time point reported						
0 months	3	15			3	3
2 months			1	1	1	1
3 months	1	5	3	4	4	4
6 months	2	10	2	3	4	4
1 year	12	60	41	55	53	56
2 years			15	20	15	16
3 years			2	3	2	2
4 years						
5 years			3	4	3	3
6–9 years			1	1	1	1
10 years or more			1	1	1	1
Total FU time not defined (e.g., continuous annual or visit-based FU)	2	10	5	7	7	7

*PROM* Patient-Reported Outcome Measure, *NR* Not reported, Percentages rounded to the nearest integer

The total number of surveys ranged from 1 (21%) to ≥ 10 (1%), most commonly two (36%). The total FU time ranged from 0 months to 15 years, with one year being most frequent (56%).

In cross-sectional datasets, typically one (70%) and in longitudinal datasets one (26%), two (34%), or three (23%) questionnaires per survey were distributed. Surveys were mostly administered via paper (22%), electronic (16%), or both (16%). Fully electronic methods appeared only in studies published from 2020 onward. Reminder use was poorly reported, with data available for only 25% of the datasets at BL and 33% at FU, most often indicating no reminders at BL and one at the last FU.

### Reporting the eligible population and response rate

The eligible population was classified as “all treated”, only in 19 out of 94 cohorts (20%). Additionally, five (5%) publications, where the coverage of recruited patients was 100% of the target population [7, 19, 22–24], were classified as all treated (Table 5).

**Table 5:** Definition of the eligible population

Eligibility, how reported?	*N* = 94	Percentage (%)
“All treated”	24	
All treated	19	20
All recruited to a CQR or another specific database, coverage 100%	5	5
“All recruited”	39	
All recruited to a CQR or another specific database, coverage NR or < 100%	39	41
“Other”	31	33
BL-respondents	11	12
FU-respondents	1	1
BL- and FU respondents	5	5
Other definition of the subset of the eligible population	14	15

*CQR* Clinical Quality Registry, *NR* Not reported, Percentages rounded to the nearest integer

The most common flaw in eligibility definition was to use “all recruited” patients without reporting coverage, or when coverage was less than 100% (39/94, 41%). The “other” labelled datasets used only BL-, FU-, or BL + FU (17, 18%) data, most often from registry-based datasets with undocumented eligibility. Additionally, 14 (15%) publications described more specific inclusion frameworks.

Among the 68 publications that used data from CQRs or other specific databases, 26 (38%) reported coverage. Among these, 19 (73%) indicated ≥ 90% coverage, with only six (23%) reporting 100% coverage. (Online Resource_1, Supplement 7).

Within the “all treated” eligibility group, only 5 out of 24 datasets used all treated in RR calculations (see Table 6 and Online Resource_1, Supplement 8). Common flaws in RR calculations included using BL respondents as the denominator at FU (5), reporting RRs only for complete BL + FU respondents (4), missing RR for all time points (3), or using those who received the questionnaire as a denominator instead of the eligible population (2). In three cases (16%), RR accuracy could not be assessed, due to missing respondent numbers, and in two cases, only respondent counts were provided without RR percentages.

**Table 6:** Ranges and average response rates (RRs) according to the definition of reporting the eligible population and calculation of RRs

Eligible population	*N* = 94	RR at baseline (BL)		RR at the last follow-up (FU)	
Reporting of response rate (RR)	*n*	Range	Average	Range	Average
“All treated”	24				
RR calculated correctly	5	100	100	34–70	54
RR calculated incorrectly	14	39–99	73	26–98	66
Calculation of the RR not possible to assess	3	56–84	69	26–93	63
RR not calculated, possible to calculate	2	100 (1)	100		
“All recruited”	39				
RR calculated denominator-consistently	18	30–99	74	18–94	67
RR calculated incorrectly	14	33–97	60	1–89	62
Calculation of the RR not possible to assess	3			58–65	62
RR not calculated, possible to calculate	4	38–99 (3)	77	48 (1)	48
“Other”	31				
RR calculated denominator-consistently	9	20–100	88	12–99	62
RR calculated incorrectly	16	74–100	89	40–85	72
Calculation of the RR not possible to assess	1	72–73	73	46–53	50
RR not calculated, possible to calculate	5	53–85 (3)	71	62–78 (2)	73

RR rounded to the nearest integer; The number in parentheses denotes the corresponding sample size, if available only for a subset of the total sample

Similar issues were found in datasets where eligibility was defined as “all recruited” or “other”. In the “all recruited” group more than half (21/39, 54%) did not use their defined eligible population in RR calculations. Similarly, in the “other” group, (22/31, 71%) authors failed to apply their own eligibility definitions. Across these two groups, RRs were classified as incorrect (30/70, 43%), mainly due to incomplete eligible populations. The most common issues included the use of BL-respondents (11) or another subset of the eligible population (5) as the determinator at FU, the exclusion of patients without adequate justification (5) or the restriction of RRs to patients with complete BL + FU data (9). In four (5%) datasets, RR accuracy could not be assessed due to missing respondent counts. For nine (13%), only respondent numbers were provided without percentages (see Table 6 and Online Resource_1, Supplement 8).

Table 6 presents a summary of average RRs according to the eligibility and RR classification. There was considerable variation in RRs across studies. The range of RRs varied from 20 to 100% at BL and from 1 to 99% at the last FU timepoint reported. The average RRs of 60–80% at BL and 60–70% at the last FU timepoint were mostly documented. The systematic use of a defined eligible population was linked to a decline in average RRs over the FU.

In general, average RRs declined over time. Correctly calculated RRs declined more over the FU period and were lower at the last FU compared to incorrect or denominator-consistent calculations, which were frequently based on subsets of eligible populations. Furthermore, among patients with only complete BL + FU data, the RR at the last FU was lower than in all incorrectly calculated RR groups.

In the eligibility group “all treated”, the correctly calculated RR averages of 5 datasets declined considerably from the BL to the last FU. Although this attrition appeared substantial, it was based on a single BL dataset. The decline of the average RR in the “all recruited”/”other” groups was smaller. In some datasets the RR even increased.

When examined across three FU time groups (Online Resource_1, Supplement 9), despite the inclusion of 74 studies, the number of observations within each group was small. The highest number of observations (*n* = 47) was observed in the 1-year FU group. No marked differences in mean RRs were detected between eligibility groups at this time point. Due to the limited number of observations per eligibility group, no clear conclusions could be drawn regarding the association between reported eligibility and RRs at the 1-year FU.

## Discussion

The main finding of this review was that eligibility was correctly defined as “all treated” in only 26% of the datasets. More commonly, the eligible population was defined as “all recruited” or “other” subset of the eligible population. As the definition of the eligible population is crucial for accurate RR calculation, the proportion of datasets using the “all treated” definition was alarmingly low. Eligibility definitions directly influence the validity and accuracy of reported RRs. If eligible individuals are excluded, the RR no longer represents the target population, an important consideration when such data are used for research or quality assessment purposes.

When eligibility was defined as “all treated”, RRs were correctly calculated in only 5 datasets. Thus, accurately according to the definitions in the scientific literature [14], reported studies represent only 5% of all 94 datasets. Even those who correctly defined the eligible population rarely used it in RR calculations consistently over time. When the authors had defined their eligible population as “all recruited” or “other”, they seldom used that population to calculate the RR. In all three eligibility groups, the main reasons for inconsistent calculation of RRs (44, 47%) included the use of incomplete populations as a denominator, often because FU questionnaires were distributed only to prior respondents or the RR was limited to patients with full BL + FU data. As a result, this led to potentially biased RRs. Additional inaccuracies arose from missing reporting of FU time points or unjustified patient exclusions.

Across publications, RRs varied widely from 20 to 100% at BL and 1–99% at the last FU. Average RRs, 60–80% at BL and 60–70% at the last FU, typically showing a downward trend over time, were consistent with findings from previous reviews [8, 10]. Despite the large number of studies included, only five calculated RRs correctly, limiting the ability to assess how calculation methods influenced the results. The only consistent finding was a greater decline in RRs from the BL to the last FU in studies using “all treated” definition compared with studies that calculated RRs incorrectly due to the use of subgroups of the eligible population.

Data collection methods differ between clinical trials and routinely collected datasets, potentially affecting both data coverage and quality. While RCTs offer high-quality data, they are resource intensive. Registry-based studies allow faster data collection but often suffer from lower data quality and RRs. Prospective cohort studies typically achieve higher RRs at BL (up to 97%), although these rates decline over time [8]. Registries show lower and more variable RRs (e.g., from 72% at BL to 47% at 2–5 years) [8–11]. Loss to FU is a common issue, potentially introducing bias and reducing statistical power [12]. The thresholds for acceptable attrition vary, with ≥ 20% often considered problematic [13]. Despite lower RRs, registry data may still reflect outcome trends in large populations [21]. However, the impact of attrition depends on cohort and clinical context [25]. Targets such as ≥ 90% coverage and ≥ 80% RR remain difficult to achieve.

Most articles (74%) were published in the 2020s, coinciding with the widespread adoption of electronic collection methods; all fully electronic datasets were published between 2020 and 2025. Similarly, 70% of the studies that used CQR/database data emerged during this period. This trend reflects the growing integration of PROMs into routine care, underscoring the increasing importance of evaluating data quality, coverage and response completeness. The coverage of a CQR/database was reported in only 38% of publications, with full coverage indicated in just 9%. Thresholds for acceptable levels of unregistered patients have been defined. For example, Dutch registries consider only very few (< 2%) unregistered patients acceptable [26], whereas Australian standards require less strict (> 85%) capture for national benchmarking and > 60% for quality improvement or research purposes [27]. Mature CQRs in Nordic countries [23, 28–31] and the United States [22, 32, 33] have achieved high 97–100% coverage rates. However, patient participation is typically voluntary and requires informed consent, posing challenges to comprehensive data capture.

PROMs were predominantly used in surgical care, with procedure-specific protocols and data mostly (72%) retrospectively extracted from registries via automated electronic platforms. While these platforms enable efficient, large-scale data capture, they may compromise data completeness [21, 27, 34] and RRs [11, 35]. Combining manual and automated methods generally improves RRs [9, 36], although manual PROM collection, often used to achieve ≥ 80% FU, is rarely cost-justified. Despite lower FU (~ 50%), automated methods may still yield unbiased outcomes when BL coverage exceeds 97% [32]. The growing utilization of routinely collected PROMs data generally aims to monitor effectiveness, which requires both BL and FU data. This underscores the importance of systematically assessing and transparently reporting both data loss and the representativeness of the datasets.

To our knowledge, this review of 92 articles is the first to examine how eligible populations are defined and how response rates are calculated in routinely collected PROMs among adult patients across diverse health care contexts. Strengths include a systematic literature search, a published protocol with all changes documented, and a transparent, carefully conducted synthesis, accessible to readers, that offers novel insights beyond specific clinical settings or disease groups.

One limitation of this study is that interpretation relied on the information provided in the included articles regarding the target population, data collection and definition of coverage and RRs. As datasets were mostly orthopedic (65%) and oncologic (14%), it is important to note that they do not comprehensively reflect the routine utilization of PROMs, as they are biased toward research-intensive specialties and units. Data collection protocols were rarely described in detail, particularly when national registries are used. Additionally, attrition and reasons for lost to FU were reported inconsistently, limiting assessment of data coverage and RR accuracy. Although we had 94 datasets in the included publications, the number of datasets in categories by eligible definition and RR calculations was small. This limited the chances for reliable comparison of RRs between the categories, as results could be influenced by single observations.

## Conclusion

The routine collection of PROMs has become increasingly common. However, based on the reporting in scientific publications, challenges arise in defining the eligible population and calculating RRs. Only 5% of the studies calculated RRs correctly. In 74% of the cases, eligible populations were not all treated patients. Even when authors define the eligible population as something other than “all treated”, they often apply this definition inconsistently in RR calculations. The RR ranges varied widely. The systematic use of a defined eligible population was associated with a decline in average RRs over the FU period. This review highlights the need to improve the reporting of eligibility and RRs while using routinely collected PROMs.

## Supplementary Information

Below is the link to the electronic supplementary material.

Supplementary Material 1

Supplementary Material 2

## Data Availability

Data are available from the corresponding author upon reasonable request.
