# Supplementary material for: Eligibility definitions and response rate accuracy in studies using routinely collected PROMs: a systematic review
Source: Qual Life Res. 2026 Jun 25;35(8):217. doi: 10.1007/s11136-026-04320-x (PMC13303456; doi:10.1007/s11136-026-04320-x)
Supplement: Supplementary file 1 — Supplementary Material 1 [file 11136_2026_4320_MOESM1_ESM.pdf]

Eligibility definitions and response rate accuracy in studies using routinely collected PROMs: a systematic review
In
Quality of Life research

Johanna Laine[a,b,†] and Eila Kankaanpää[a]

a Department of Health and Social Management, University of Eastern Finland, Kuopio, Finland.
b Department of Strategy and Development, Wellbeing Services County of North Savo, Kuopio, Finland

†Corresponding author: johanna.laine@uef.fi

Online Resource_1, containing Supplements 1-9:

Supplement 1 - PRISMA Abstract checklist

Section and Topic	Item #	Checklist item	Reported (Yes/No)
**TITLE**			
Title	1	Identify the report as a systematic review.	Yes
**BACKGROUND**			
Objectives	2	Provide an explicit statement of the main objective(s) or question(s) the review addresses.	Yes
**METHODS**			
Eligibility criteria	3	Specify the inclusion and exclusion criteria for the review.	Yes
Information sources	4	Specify the information sources (e.g. databases, registers) used to identify studies and the date when each was last searched.	Yes
Risk of bias	5	Specify the methods used to assess risk of bias in the included studies.	NA
Synthesis of results	6	Specify the methods used to present and synthesise results.	Yes

Section and Topic	Item #	Checklist item	Reported (Yes/No)
**RESULTS**			
Included studies	7	Give the total number of included studies and participants and summarise relevant characteristics of studies.	Yes
Synthesis of results	8	Present results for main outcomes, preferably indicating the number of included studies and participants for each. If meta-analysis was done, report the summary estimate and confidence/credible interval. If comparing groups, indicate the direction of the effect (i.e. which group is favoured).	Yes
**DISCUSSION**			
Limitations of evidence	9	Provide a brief summary of the limitations of the evidence included in the review (e.g. study risk of bias, inconsistency and imprecision).	NA
Interpretation	10	Provide a general interpretation of the results and important implications.	Yes
**OTHER**			
Funding	11	Specify the primary source of funding for the review.	Funding is reported in Statements and Declarations according to the journal's guidelines.
Registration	12	Provide the register name and registration number.	Yes

Supplement 2 - PRISMA checklist

Section and Topic	Item #	Checklist item	Location where item is reported
**TITLE**			
Title	1	Identify the report as a systematic review.	Title page
**ABSTRACT**			
Abstract	2	See the PRISMA 2020 for Abstracts checklist.	Abstract (p. 2)
**INTRODUCTION**			
Rationale	3	Describe the rationale for the review in the context of existing knowledge.	Introduction (p. 3)
Objectives	4	Provide an explicit statement of the objective(s) or question(s) the review addresses.	Specific aims and main outcome(s) (p. 3)
**METHODS**			
Eligibility criteria	5	Specify the inclusion and exclusion criteria for the review and how studies were grouped for the syntheses.	Inclusion and exclusion criteria (p. 3-4), Table 1 (p.4), and Online resource_1 (Supplement 3)
Information sources	6	Specify all databases, registers, websites, organisations, reference lists and other sources searched or consulted to identify studies. Specify the date when each source was last searched or consulted.	Abstract; Search strategy and screening, (p. 4-5) and Online Resource_1 (Supplement 4)
Search strategy	7	Present the full search strategies for all databases, registers and websites, including any filters and limits used.	Search strategy and screening (p. 4-5) and Online Resource_1 (Supplement 4)
Selection process	8	Specify the methods used to decide whether a study met the inclusion criteria of the review, including how many reviewers screened each record and each report retrieved, whether they worked independently, and if applicable, details of automation tools used in the process.	Data extraction (p. 5) and PRISMA flow diagram, Fig 1 (p. 6)
Data collection process	9	Specify the methods used to collect data from reports, including how many reviewers collected data from each report, whether they worked independently, any processes for obtaining or confirming data from study investigators, and if applicable, details of automation tools used in the process.	Search strategy and screening (p. 4-5), Data extraction (p. 5), and Data synthesis, (p. 5)

Section and Topic	Item #	Checklist item	Location where item is reported
Data items	10a	List and define all outcomes for which data were sought. Specify whether all results that were compatible with each outcome domain in each study were sought (e.g. for all measures, time points, analyses), and if not, the methods used to decide which results to collect.	Data synthesis (p. 5-6) and Online Resource_2 (Data extraction sheet)
	10b	List and define all other variables for which data were sought (e.g. participant and intervention characteristics, funding sources). Describe any assumptions made about any missing or unclear information.	Data synthesis (p. 5-6), Online Resource_1 (Supplement 5), and Online Resource_2 (Data extraction sheet)
Study risk of bias assessment	11	Specify the methods used to assess risk of bias in the included studies, including details of the tool(s) used, how many reviewers assessed each study and whether they worked independently, and if applicable, details of automation tools used in the process.	Search strategy and screening (p. 4-5), Data extraction (p. 5), and Discussion (p. 13)
Effect measures	12	Specify for each outcome the effect measure(s) (e.g. risk ratio, mean difference) used in the synthesis or presentation of results.	Data synthesis (p. 5-6)
Synthesis methods	13a	Describe the processes used to decide which studies were eligible for each synthesis (e.g. tabulating the study intervention characteristics and comparing against the planned groups for each synthesis (item #5)).	Data synthesis (p. 5-6)
	13b	Describe any methods required to prepare the data for presentation or synthesis, such as handling of missing summary statistics, or data conversions.	Data synthesis (p. 5-6)
	13c	Describe any methods used to tabulate or visually display results of individual studies and syntheses.	Data synthesis (p. 6)
	13d	Describe any methods used to synthesize results and provide a rationale for the choice(s). If meta-analysis was performed, describe the model(s), method(s) to identify the presence and extent of statistical heterogeneity, and software package(s) used.	Data synthesis (p. 5-6)
	13e	Describe any methods used to explore possible causes of heterogeneity among study results (e.g. subgroup analysis, meta-regression).	Data synthesis (p. 6)
	13f	Describe any sensitivity analyses conducted to assess robustness of the synthesized results.	Data synthesis (p. 6), Online Resource_1 (Supplement 9)
Reporting bias assessment	14	Describe any methods used to assess risk of bias due to missing results in a synthesis (arising from reporting biases).	The aim of the review was to examine potential bias in original

Section and Topic	Item #	Checklist item	Location where item is reported
			authors reported eligibility definitions and RR calculations. The review assesses the quality of how RRs are calculated and reported.<br>For example: Inclusion and exclusion criteria (p. 3-4), Table 6 (p. 11), Online Resource_1 (Supplement 9) and Online Resource_2 (Data extraction sheet)
Certainty assessment	15	Describe any methods used to assess certainty (or confidence) in the body of evidence for an outcome.	The aim of the review was to examine potential bias in original authors reported eligibility definitions and RR calculations. The review assesses the quality of how RRs are calculated and reported.
**RESULTS**			
Study selection	16a	Describe the results of the search and selection process, from the number of records identified in the search to the number of studies included in the review, ideally using a flow diagram.	Results (p. 6-7) and PRISMA flow diagram (p. 6)
	16b	Cite studies that might appear to meet the inclusion criteria, but which were excluded, and explain why they were excluded.	The reasons for exclusion were recorded in Covidence and are presented in PRISMA flow diagram (p. 6)
Study characteristics	17	Cite each included study and present its characteristics.	Online resource_2 (Data extraction sheet) and Table 2 (p. 7-8)
Risk of bias in studies	18	Present assessments of risk of bias for each included study.	Online resource_2 (Data extraction sheet, columns AA-AG)
Results of individual studies	19	For all outcomes, present, for each study: (a) summary statistics for each group (where appropriate) and (b) an effect estimate and its precision (e.g. confidence/credible interval), ideally using structured tables or plots.	Online resource 2 (Data extraction sheet)

Section and Topic	Item #	Checklist item	Location where item is reported
Results of syntheses	20a	For each synthesis, briefly summarise the characteristics and risk of bias among contributing studies.	Reporting the eligible population and response rate (p. 10-11) and Discussion (p. 11-12), Online Resource_2
	20b	Present results of all statistical syntheses conducted. If meta-analysis was done, present for each the summary estimate and its precision (e.g. confidence/credible interval) and measures of statistical heterogeneity. If comparing groups, describe the direction of the effect.	NA
	20c	Present results of all investigations of possible causes of heterogeneity among study results.	Reporting the eligible population and response rate (p. 10-11) and Discussion (p. 11-12)
	20d	Present results of all sensitivity analyses conducted to assess the robustness of the synthesized results.	Reporting the eligible population and response rate (p. 10-11), Online Resource_1 (Supplement 9)
Reporting biases	21	Present assessments of risk of bias due to missing results (arising from reporting biases) for each synthesis assessed.	The aim of the review was to examine potential bias in original authors reported eligibility definitions and RR calculations. The review assesses the quality of how RRs are calculated and reported.
Certainty of evidence	22	Present assessments of certainty (or confidence) in the body of evidence for each outcome assessed.	The aim of the review was to examine potential bias in original authors reported eligibility definitions and RR calculations. The review assesses the quality of how RRs are calculated and reported.
**DISCUSSION**			
Discussion	23a	Provide a general interpretation of the results in the context of other evidence.	Discussion (p. 12-13)
	23b	Discuss any limitations of the evidence included in the review.	Discussion (p. 12-13)
	23c	Discuss any limitations of the review processes used.	Discussion (p. 12-13)

Section and Topic	Item #	Checklist item	Location where item is reported
	23d	Discuss implications of the results for practice, policy, and future research.	Conclusion (p. 13)
**OTHER INFORMATION**			
Registration and protocol	24a	Provide registration information for the review, including register name and registration number, or state that the review was not registered.	Abstract (p. 2) and Methods (p. 3)
	24b	Indicate where the review protocol can be accessed, or state that a protocol was not prepared.	Methods (p. 3)
	24c	Describe and explain any amendments to information provided at registration or in the protocol.	Online resource_1 (Supplement 5)
Support	25	Describe sources of financial or non-financial support for the review, and the role of the funders or sponsors in the review.	Reported in Statements and Declarations according to the journal's guidelines.
Competing interests	26	Declare any competing interests of review authors.	Reported in Statements and Declarations according to the journal's guidelines.
Availability of data, code and other materials	27	Report which of the following are publicly available and where they can be found: template data collection forms; data extracted from included studies; data used for all analyses; analytic code; any other materials used in the review.	All articles included in this review are publicly available. A detailed description of the data extraction is provided in Online Resource_2. The extracted data are available from the corresponding author upon reasonable request.

From: Page MJ, McKenzie JE, Bossuyt PM, Boutron I, Hoffmann TC, Mulrow CD, et al. The PRISMA 2020 statement: an updated guideline for reporting systematic reviews. BMJ 2021;372:n71. doi: 10.1136/bmj.n71. This work is licensed under CC BY 4.0. To view a copy of this license, visit https://creativecommons.org/licenses/by/4.0/

Supplement 3 – The modified criteria for the inclusion and exclusion of the articles

Context:

1. If PROMs data collection was related to non-healthcare context, such as: general or normative population, community dwelling, (professional or former) athletes, sports, military; or other settings not related to the collection of PROMs in the routine daily processes of health care, such as global pandemic (COVID-19, COVAD, COVID vaccination), article was excluded.

2. PROMs providing only general health information, for example performance-based tests like "the 6 minutes walking test", article was excluded.

3. Routine collection of PROMs was defined as the adoption and use of PROMs within real-world health care settings. It involves the systematic capture of PROM data under everyday clinical conditions as part of regular care processes and follow-up.

- PROMs data collected routinely as part of the data collection of clinical quality registers (CQRs), was considered routine collection of the PROMs.
- If prospectively and routinely collected PROMs data was retrospectively reviewed in cohort studies, case studies, case series or in cross-sectional studies, and the focus was the PROMs data used in healthcare context, they were included. Articles whose focus was not related to the health care context were excluded. If the primary focus of the study was not directly related to the RR of routinely collected PROMs data, the article was not excluded automatically. Appropriateness for inclusion was assessed on a case-by-case basis to ensure consistency with the review's inclusion criteria. Decisions regarding inclusion or exclusion were made accordingly.
- Case studies and case reports containing routinely collected PROMs reported by a single respondent (e.g. a patient or a surgeon) were excluded.
- Short-term postoperative remote monitoring questionnaires (e.g. Recovery Tracker used in ambulatory surgical care) were considered to reflect treatment monitoring rather than the evaluation of PROMs and were therefore excluded.
- Studies conducted in non-health care context or not related to the routine collection of PROMs such as trials, pharmaceutical studies or for specific research purposes were excluded.
- If prospectively and routinely collected PROMs data was retrospectively reviewed and (additional) PROMs were collected in study settings from limited, for the research purposes recruited population, article was excluded.
- Review articles, dissertations, letters to editor, editorial or other commentaries, handbooks and book chapters were excluded, because these publication types do not typically provide primary data, methodological details, or outcome-specific information required for systematic evaluation. As such, they do not meet the inclusion criteria for primary research and are not suitable resources for response rate extraction or analysis.
- Feasibility- and development studies (related to: validation, psychometric analysis, development of frameworks, anchor questions/-methods, definition of MICs/MIDs thresholds, crosswalks, response shift/recalibration, comparisons/evaluations of suitability of PROM measures) as well as pilots, development projects, short-term or limited-duration research initiatives, as well as PROMs collected within clinical trials or real-world evidence (RWE) pharmaceutical studies, were not considered to represent routine PROM collection or the use of routinely collected PROMs data in health care decision-making.

- Articles containing information only on the experience of care received, satisfaction with care or (pain)medication, and/or feedback for health care providers, personnel, or services, were excluded.

Phenomena of interest:

4. If the response rate (RR) and the size of the target population (i.e., the total number of eligible patients) were not reported in the abstract, the full text was reviewed. Because the research question specifically focused on how authors defined eligible populations and calculated RRs of routinely collected PROMs, articles were excluded if the number of eligible population and/or RRs were not reported by original authors, or these values could not be retrieved from the full text and be calculated by review. Without this information, the article could not contribute evidence relevant to answering the research question.

Concept/administration

6. PROMs reported by health care professionals (HCP), proxy (relative or other caregiver/contact person acting on behalf of the patient) with no input from the patient were excluded.

- In the case of the child being the object of the treatment, if proxy-reported from the parents' or other caregivers' point of view, articles were excluded.

Population:

7. Studies in which less than 10% of the study population were 17 years and younger (children, pediatric, adolescents etc.), if the other inclusion criteria were met, the article was included. If more than 10 % of the patients were 17 years or younger, the article was excluded.

Supplement 4 - Search strategy

Table S1 Search terms by database

Database	Search terms
**PubMed**	(((Patient reported Outcome Measures[MeSH Terms]) OR ("patient reported outcome*"[Title/Abstract] OR PROM[Title/Abstract] OR PROMs[Title/Abstract] OR ePROM[Title/Abstract] OR "patient outcome assessment"[Title/Abstract])) AND ("response rate*"[Title/Abstract] OR "response activit*"[Title/Abstract] OR"non respon*"[Title/Abstract] OR "nonrespon*"[Title/Abstract] OR "not respon*"[Title/Abstract])) NOT (trial*[Title/Abstract] OR non-RCT*[Title/Abstract])
**Scopus**	(TITLE-ABS-KEY("patient reported outcome measure*" OR "patient reported outcome*" OR PROM OR PROMs OR ePROM OR "patient outcome assessment*") AND TITLE-ABS-KEY("response rate*" OR "response activit*" OR "non respon*" OR "nonrespon*" OR "not respon*") AND NOT TITLE-ABS-KEY(trial* OR RCT* OR non-RCT*)
**Web of Science**	(AB=("Patient Reported Outcome Measure*" OR "patient reported outcome*" OR PROM OR PROMs OR ePROM OR "patient outcome assessment*")) AND AB=("response rate*" OR "response activit*" OR "non respon*" OR "nonrespon*" OR "not respon*") NOT (AB=(trial* OR RCT* OR non-RCT*)
**CINAHL and PsycInfo**	AB ("Patient reported Outcome Measure*" OR "patient reported outcome*" OR PROM OR PROMs OR ePROM OR "patient outcome assessment*") AND AB ("response rate*" OR "response activit*" OR "non respon*" OR "nonrespon*" OR "not respon*") NOT AB (trial* OR RCT* OR non-RCT*)
**Cochrane**	#1 ("Patient Reported Outcome Measure" OR "patient reported outcome" OR PROM OR PROMs OR ePROM OR "patient outcome assessment"):ti,ab,kw<br>#2 MeSH descriptor: [Patient Reported Outcome Measures] explore all trees<br>#3 ("response rate" OR "response activity" OR "non response" OR "nonresponse"):ti,ab,kw<br>#4 (#1 OR #2)<br>#5 (#4 AND #3)<br>in Cohrane reviews, Cochrane Protocols, Clinical Answers, Editorials and Special Collections

Supplement 5 - The data extraction tool with modifications

**Study Details**	**Modifications**
Author, first	
Year of publication	
The study period [year/month]_[year/month]	Added
Country of origin	
Title of the article	
Aim/purpose of the study	
Study design	
Method(s)	Removed
Outcome measures	Removed
Population	
Diagnostic group to whom PROM was developed and applied to	Removed
**Context of use**	
Clinical setting (health care):	Added: type of organization or facility, type of care
Organizational level of the Patient-reported Outcome Measure (PROM) data collection	Added
Details of the Clinical Quality Registry (CQR) or other clinical (specific) database if used for PROMs data collection	Added
The level of the CQR (local, national, international) if applicable	Added
The coverage of the CQR: reported (yes/no); captured of the eligible population (%)	Added
**Concept/Interest:**	
The name(s) of the PROM(s)	Removed the version of the PROM(s)
The purpose of the PROM: intended use, actual use	Removed
Number of items	Removed
Response time/time to complete the measure (minutes)	Removed
Start year of PROM collection	Removed
Details of the routine collection process of the PROMs data	
Mode of administration	Removed

Method(s) of administration of the PROM surveys	Added classification of data collection methods: paper, electronic, telephone, else and combinations of these
Location of administration (e.g. clinic, home, other)	Removed
Recall timeframe	Modified: Timing of PROM surveys (point-of-care, POC)
The number of questionnaires per timepoint(s) reported in the study	Added
The number of items per time point	Removed
Number of recalls	Modified: The total number of surveys
The total follow-up (FU) time (months, years)	Added months
The number of reminders (per time point)	Modified, type of reminders removed
**Reporting of the eligible and RRs**	
Definition of the eligible (target population)	Added
Sample size	Added the number of the eligible
The number of the subset of the eligible population that was included in the study	Added
The response rate (RR) of the first (baseline, BL) survey and the last (FU) survey reported in the study, if available (%)	Added specified PROMs measurement time points: the first at baseline and the last at the last follow-up time point
Methods used to calculate RR(s)	
Completion rate, item response (%)	Removed
Refusal rate (%)	Removed
Number and details of missing data	Removed
Assessment of missing data (Is potential nonresponse bias assessed?)	Removed
Incentives used to increase responsiveness and/or representativeness of the data (e.g., monetary, non-monetary)	Removed
Integration with electronic health records (EHRs)	Removed
How the data is reported (scoring)	Removed
Feedback to (HCP/patient/both)	Removed
Content of feedback (e.g. scores)	Removed
Education for Health care personnel (HCP), interpretation of the PROM results	Removed
Considerations of the quality of the PROMs data	Removed
Results/key findings	
Comments	

Supplement 6 - The list of acronyms and corresponding full terms of the PROM questionnaires

Acronym of the PROM questionnaire	Full term of the PROM questionnaire
15D	15D-dimensional instrument
AFSS	Atrial Fibrillation Severity Scale (duration, frequency, severity)
ASES	American Shoulder and Elbow Surgeons Score
AVVQ	Aberdeen Varicose Vein Questionnaire
BASDAI	Bath Ankylosing Spondylitis Disease Activity Index
BASFI	Bath Ankylosing Spondylitis Functional Index
Breast-Q	Breast Cancer modules
Charnley's functional categories A, B, C	Charnley classification's functional categories A, B, C
COMI	Core Outcome Measure Index
DASH	Disabilities of the Arm, Shoulder and Hand (30 items)
EORTC QLQ C-30	The European Organization for Research and Treatment of Cancer QLQ-30
EORTC QLQ-BR23	The European Organization for Research and Treatment of Cancer - Breast Cancer
EORTC QLQ-C15-PAL	The European Organization for Research and Treatment of Cancer - Palliative
EORTC QLQ-HN35	The European Organization for Research and Treatment of Cancer - Head and Neck
EPH-30	Endometriosis health profile-30
EPIC-26	Expanded Prostate Cancer Composite-26
EQ-5D-3L/EQ-5D-5L	3-level/5-level version of the EuroQol Group Five dimension (EQ-5D)
EQ-VAS	EQ Visual Analogue Scale
ESAS	Edmonton Symptom Assessment Scale
FAAM-ADL	Foot and Ankle Ability Measure, Activities of Daily Living subscale
FACT-G	Functional Assessment of Cancer Therapy -General
FAOS	Foot and Ankle outcome score
FJS-12	Forgotten Joint Score-12
GA back/leg pain	Global Assessment
GTO	Global Treatment Outcome
HADS	Hospital Anxiety and Depression Scale
HHS	Harris Hips Score
HOOS	Hip disability and Osteoarthritis Outcome Score (40 items)

HOOS-JR	Hip disability and Osteoarthritis Outcome Score (six items)
HQ-8	The eight-item patient questionnaire
HUNT (physical activity)	Hunt physical activity questionnaire
IPQ	Inguinal Pain Questionnaire
KOOS	Knee Injury and Osteoarthritis Outcome Score (42 items)
KOOS-JR	Knee Injury and Osteoarthritis Outcome Score (seven items)
KSS	Karolinska Sleepliness Scale
MDHAQ	Multidimensional Health Assessment Questionnaire
mJOA	Modified Japanese Orthopaedic Association (mJOA) score
MYMOP	Measure Yourself Medical Outcome Profile
NASS-LS	North American Spine Society Low Back Instrument
NDI	Neck Disability Index
NPS	Net Promoter Score
NRS	Numeric rating scale
ODI	Oswestry Disability Index
OHS	Oxford Hip Score
OKS	Oxford Knee Score
PASS	Patient Acceptable Symptom scale
PGA	Patient's Global Assessment Scale
PGIC	Patient's Global Impression of Change
PROMIS	Patient-Reported Outcomes Measurement Information System
Prostate QoL survey	Prostate Quality of Life survey
PSCI	Prostate Cancer Index
PSFS	Patient Spesific Functional scale
PSS	Pain Severity Scale
QuickDASH	Disabilities of the Arm, Shoulder and Hand (11 items)
RAND12	RAND 12 Item Health Survey
SANE	Single Assessment Numeric Scale
SAS	Self-Rating Anxiety Scale
SEFAS	Self-reported Foot and Ankle Score
SF-12	12-item Short Form Survey
SF-36	36-Item Short Form Survey

SLAG questionnaire (33 items)	
SMFA (dysfunction index, bother index)	Short Musculoskeletal Function Assessment
SRH (from SF-36)	Self-Rated Health, item 1 of the SF-36
SST	Simple Shoulder Test
SWB	Subjective Well-Being Scale
the HeartQoL	HeartQoL Health-Related Quality of Life Questionnaire
the MacNew Heart	MacNew Heart Disease Health-related Quality of Life Questionnaire
The Memorial Sloan Kettering Cancer Center tool	
UCLA activity score	University of California, Los Angeles (UCLA) Activity Score
VAS	Visual Analogue Scale
VR-12	The Veterans RAND 12-item Health Survey
WHODAS	World Health Organization Disability Assessment Schedule
WOMAC	Western Ontario and McMaster Universities Osteoarthritis Index
WOOS	Western Ontario Osteoarthritis of the Shoulder Index
WORC	Western Ontario Rotator Cuff Index
WOSI	Western Ontario Shoulder Instability Index

Supplement 7 - The coverage of the CQR or another specific PROM database (N = 68)

Coverage[a] (%)	The number of study cohorts (n)	Portion of total (%)
100	6	9
98-99	9	13
90-97	4	6
80-89	0	0
60-79	6	9
1-59	1	1
NR	42	62
All	68	100

[a]Coverage = The proportion of eligible population registered into the clinical Quality Registry (CQR) or another specific PROM database; *NR* coverage not reported

Supplement 8 - Assessment of RR calculations, across the defined eligibility groups (N = 94 datasets)

Classification of the eligible population and response rates (RRs)	All treated (n)	All treated (%)	Other (n)	Other (%)
**Eligible population reported as "all treated"**	**24**	**26**		
**RR calculated correctly**	**5**	5		
**RR calculated incorrectly**	**14**	15		
RR at FU reported for the BL respondents (not for all eligible)	5			
RR reported only for the patients with complete PROMs data at both BL and FU (study group)	4			
RR reported only for the patients who had answered PROMs at least once at some FU time point (RR at FU timepoints NR) or RR at data collection FU time points NR	3			
RR at FU reported for those to whom the PROMs had been administered (not for all eligible)	2			
**Calculation of the RRs not possible to assess**	**3**	3		
Only RR percentages at BL and or FU time points were reported, with no accompanying number of respondents	3			
**RR not calculated by original authors**	**2**	2		
Only the number of respondents at BL and or FU time points was reported, RR percentages were not provided	2			
**Eligible population reported as "all recruited" or "other"**			**70**	**74**
**RR calculated denominator-consistently**			**27**	29
**RR calculated incorrectly**			**30**	32
RR at FU reported for the BL respondents (not for all eligible)			11	
RR reported only for patients with complete PROMs data at both BL and FU (study group)			9	
RR at FU reported for a subgroup (not for all eligible), incorrect reason for exclusion or reasons for exclusion NR			5	
RR at FU reported for another subgroup (not for all eligible)			5	
**Calculation of the RRs not possible to assess**			**4**	4
Only RR percentages were reported, with no accompanying number of respondents			4	
**RR not calculated by original authors**			**9**	10
Only the number of respondents at BL and or FU timepoints was reported, RR percentages were not provided			9	

Supplement 9 - Average RRs at the last follow-up across three follow-up time groups (N = 74)

The last follow-up timepoint	BL	BL	The last FU	FU <1y	FU 1y	FU >1y
	N = 94		N = 74	n = 9	n = 47	n = 18
**Reporting of response rate (RR)**	**n**	**%**	**n**	**%**	**%**	**%**
**"All treated"**	**24**		**17**			
RR calculated correctly	5	100	5	70	65	57
RR calculated incorrectly	14	73	9	76	60	56
Calculation of the RR not possible to assess	3	69	3		55	67
RR not calculated, possible to calculate	2	100				
**"All recruited"**	**39**		**34**			
RR calculated denominator-consistently	18	74	17		61	78
RR calculated incorrectly	14	60	14	83	59	62
Calculation of the RR not possible to assess	3		2			64
RR not calculated, possible to calculate	4	77	1		48	
**"Other"**	**31**		**23**			
RR calculated denominator-consistently	9	88	6	44	63	99
RR calculated incorrectly	16	89	15	77	71	82
Calculation of the RR not possible to assess	1	73	1		50	
RR not calculated, possible to calculate	5	71	1			62

FU Follow-up; Percentages are rounded to the nearest integer; [a)] At the last follow-up time point (N = 74), datasets were excluded if the time point of the last FU was not reported, if the RR was not reported at the last follow-up, or if the correctness of the RR calculation could not be assessed.
